# ECG monitoring of post-stroke occurring arrhythmias: an observational study using 7-day Holter ECG

**DOI:** 10.1038/s41598-021-04285-6

**Published:** 2022-01-07

**Authors:** Claudia Carrarini, V. Di Stefano, M. Russo, F. Dono, M. Di Pietro, N. Furia, M. Onofrj, L. Bonanni, M. Faustino, M. V. De Angelis

**Affiliations:** 1 Department of Neuroscience, Imaging and Clinical Sciences, University G. d’Annunzio of Chieti-Pescara, Chieti, Italy; 2 Department of Biomedicine, Neuroscience and Advanced Diagnostic (BIND), University of Palermo, Palermo, Italy; 3 Department of Cardiology and Cardiac Surgery, University G. d’Annunzio of Chieti-Pescara, Chieti, Italy; 4 Department of Medicine and Aging Sciences, University G. d’Annunzio of Chieti-Pescara, Chieti, Italy; 5 Department of Neurology, “SS Annunziata” Hospital, Chieti, Italy

**Keywords:** Cardiology, Health care, Neurology

## Abstract

Post-stroke arrhythmias represent a risk factor for complications and worse prognosis after cerebrovascular events. The aims of the study were to detect the rate of atrial fibrillation (AF) and other cardiac arrhythmias after acute ischemic stroke, by using a 7-day Holter ECG which has proved to be superior to the standard 24-h recording, and to evaluate the possible association between brain lesions and arrhythmias. One hundred and twenty patients with cryptogenic ischemic stroke underwent clinical and neuroimaging assessment and were monitored with a 7-day Holter ECG. Analysis of the rhythm recorded over 7 days was compared to analysis limited at the first 24 h of monitoring. 7-day Holter ECG detected AF in 4% of patients, supraventricular extrasystole (SVEB) in 94%, ventricular extrasystole (VEB) in 88%, short supraventricular runs (SVRs) in 54%, supraventricular tachycardia in 20%, and bradycardia in 6%. Compared to the first 24 h of monitoring, 7-Holter ECG showed a significant higher detection for all arrhythmias (AF p = 0.02; bradycardia p = 0.03; tachycardia p = 0.0001; SVEB p = 0.0002; VEB p = 0.0001; SVRs p = 0.0001). Patients with SVRs and bradycardia were older (p = 0.0001; p = 0.035) and had higher CHA_2_DS_2_VASc scores (p = 0.004; p = 0.026) respectively, in the comparison with patients without these two arrhythmias. An association was found between SVEB and parietal (p = 0.013) and temporal (p = 0.013) lobe lesions, whereas VEB correlated with insular involvement (p = 0.002). 7-day Holter ECG monitoring proved to be superior as compared to 24-h recording for the detection of all arrhythmias, some of which (SVEB and VEB) were associated with specific brain areas involvement. Therefore, 7-day Holter ECG should be required as an effective first-line approach to improve both diagnosis and therapeutic management after stroke.

## Introduction

Cardiac complications, such as arrhythmias, congestive heart failure, and myocardial injuries, are fairly frequent after acute stroke^1,2^. New-onset arrhythmias, including supraventricular tachycardia, supraventricular extrasystole (SVEB), ventricular extrasystole (VEB), bradycardia, atrial fibrillation (AF) and QT interval prolongation, tend to occur often during the first few hours after stroke^3^. Although preexisting cardiac pathologies may worsen clinical outcomes^4^, arrhythmias are common in stroke patients even in the absence of previous heart diseases, suggesting also a role for central nervous system (CNS) in these abnormalities^2,5^.

However, rhythmic disturbances may also lead to brain ischemia. AF represents a major cause of approximately 30% of strokes^6^ and, because of its possible paroxysmal pattern, it could be underdiagnosed^7^.

A strict interaction between cardiovascular and neurological systems is well known^8^. As cardioembolism is a common cause of stroke, acute cerebral ischemia itself seems to induce an imbalance of central autonomic control, leading then to cardiac dysfunctions. The sympathetic and parasympathetic imbalance after stroke is documented by impairment of heart rate and blood pressure regulation^9^ and by an increased catecholamine release^10^, which mediates beta-adrenergic effects on the myocardium. Moreover, the significant increase of cardiac enzymes following acute stroke enhances the evidence of an interplay between stroke and cardiac muscle disturbances^11^.

Previous studies^12,13^ suggested also that the brain cortex exerts a control of the autonomic system, therefore specific cerebral areas involved in the ischemic damage might contribute to arrhythmogenesis.

As a result, current recommendations suggest a prolonged cardiac monitoring after stroke in order to detect arrhythmias^14^. Despite recent studies^15^ reported higher sensitivity of continuous electrocardiographic (ECG) recording, using either external/implantable loop recorders^16^ or long-term Holter ECG^17^, as compared to 24-h Holter monitoring, those methods are still not widely used. Currently, among all these methods, implantable loop recording has shown the highest sensitivity in arrhythmias detection, especially AF^18–20^. An early and effective identification of cardiac disturbances is required for treatment management to prevent morbidity and mortality after acute stroke.

The aim of the present study was to detect the rate of both AF and other cardiac arrhythmias after stroke. A further sub-analysis was also carried out to confirm the advantage of using 7-day Holter ECG, which has been suggested to be more accurate than 24-h recordings for arrhythmias detection.

A second aim was to evaluate a possible correlation between cerebral ischemic areas and specific arrhythmia occurrence to support the hypothesis of a role for CNS in arrhythmogenesis.

## Methods

One hundred and twenty patients with cryptogenic stroke^21^, referred to the Neurology Clinic of “SS Annunziata” Hospital of Chieti from July 2018 to March 2020 for ischemic stroke, were included in the study. Exclusion criteria were a previous diagnosis of AF (or at the admission with a standard ECG exam), known etiology of stroke, and ongoing anticoagulation therapy.

The study was performed according to the declaration of Helsinki and its later amendments, and it was approved by the ethical committee of the Department of Neuroscience, Imaging and Clinical Sciences of Chieti. A written consent for research purposes was obtained from all patients.

### Clinical and diagnostic assessment

Each patient underwent a complete neurological examination and the “National Institutes of Health Stroke Scale" (NIHSS)^22^ was estimated both at admission and discharge.

At admission, a thorough medical history was collected, including cardiovascular risk factors such as hypertension, diabetes, smoking, heart failure, and previous coronary disease or transient ischemic attacks/strokes. A CHA_2_DS_2_VASc score was calculated. Blood test with lipid profile was performed for each patient, whereas thrombophilia screening was done only in those younger than 60 years.

All patients underwent carotid doppler ultrasonography to evaluate the presence of extracranial atherosclerosis (< 50% if in the same vascular territory of the infarct or ≥ 50% if in a different territory from the infarct), and brain Computed Tomography. Sixty-two patients also underwent a brain Magnetic Resonance Imaging to better characterize ischemic lesions.

Considering patient history, only eight subjects performed a Transcranial Doppler Sonography to investigate the presence of patent foramen ovale.

Sixty-two patients underwent a Transthoracic Echocardiography (TTE) and left atrial volume index (LAVI) and ejection fraction were estimated. LAVI, calculated by dividing left atrial volume by body surface area, was classified as follows: normal (16–28 ml/m^2^), mildly abnormal (29–33 ml/m^2^), moderately abnormal (34–39 ml/m^2^), and severely abnormal (≥ 40 ml/m^2^). The other fifty-eight patients performed a TTE in a different Center after hospital discharge. These exams were not considered in the study because of missing data (e.g., LAVI measurements).

Within one week after the admission, all patients were monitored with a 7-day continuous Holter ECG recording.

### 7-day Holter ECG monitoring

7-day Holter ECG (AthenaDiax, Medtronic)^23^, a continuous ECG recording device, is a small and non-invasive body sensor composed of two devices, an ECG recorder (ARES) and an ECG patch (ZEUS 7D). ARES records heart rhythm, detecting episodes of AF (of at least 30 s duration), bradycardia (< 50 beats for minute, bpm), supraventricular tachycardia (> 109 bpm), SVEB, short supraventricular runs (SVRs, ≥ 120 bpm, 3 beats = 1 run), VEB, and cardiac pauses (> 3000 ms, ms). ZEUS 7D is a single-use product located on the patient’s chest. After recording, ARES is placed into the reader (IRIS), which is connected to a PC via USB cable. Data were analyzed using ThemisLight Software (v2.015; https://www.athenadiax.de/index.php?option=com_content&view=article&id=54:themis-italienisch&catid=13&lang=it&Itemid=264)^23^. All data were manually inspected and validated by an expert cardiologist.

### Statistical analysis

Categorical variables were reported as absolute numbers and percentages. Distributed continuous variables were reported as mean ± standard deviation (SD). Categorical variables were compared using ANOVA, continuous variables using the Mann–Whitney U test. Correlations between variables were assessed by means of the Spearman’s correlation coefficient.

All tests were performed using SPSS Statistic (v26) and the level of significance was set at 0.05.

## Results

### Study population

Demographic characteristics of the study sample are described in Table 1. The most frequent risk factors for stroke were hypercholesterolemia (62% of patients), hypertension (60%), diabetes (35%), and carotid stenosis (20%), including either < 50% of stenosis in the same vascular territory as the infarct, or ≥ 50% if in a different vascular territory.

**Table 1:** Demographic data, vascular risk factors and stroke severity.

	Total (n = 120)
Age, years (mean ± SD)	69,5 ± 14,0
Male, n (%)	66 (55%)
CHA_2_DS_2_VASc score (mean ± SD)	4.9 ± 1.5
Hypertension, n (%)	74 (60%)
Diabetes, n (%)	43 (35%)
Smoking, n (%)	10 (8%)
Hypercholesterolemia, n (%)	76 (62%)
Chronic heart failure, n (%)	9 (7%)
Echocardiography: EF (%, mean ± SD)	60.7 ± 7.4
Coronary artery disease, n (%)	15 (12%)
Carotid stenosis^a^, n (%)	24 (20%)
Previous stroke, n (%)	14 (11%)
Baseline NIHSS score (mean ± SD)	4.4 ± 2.9
Discharge NIHSS score (mean ± SD)	2.4 ± 2.5

*n* number of patients, *SD* standard deviation, *CHA*_*2*_*DS*_*2*_*VASc* score assessing risk of atrial fibrillation, *EF* cardiac ejection fraction, *NIHSS* National Institutes of Health Stroke Scale.

^a^Carotid stenosis is considered as < 50% or ≥ 50% if in a different vascular territory.

At the admission, systemic fibrinolytic therapy was performed in 23 patients (19%).

### Arrhythmic events

**Figure 1:**
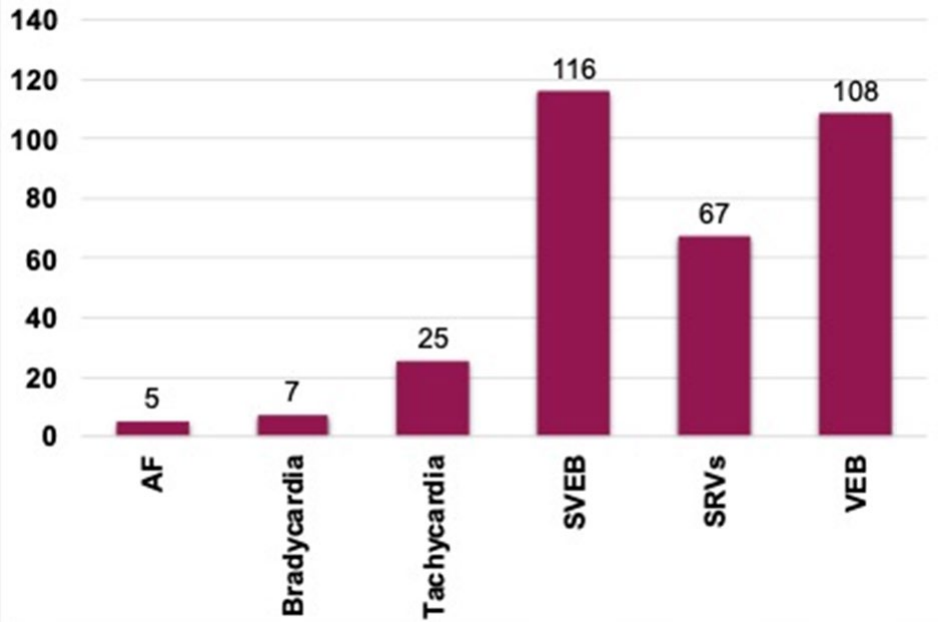
The rate of detection for all arrhythmias with 7-day Holter ECG. The ordinate represents the number of episodes detected for each arrhythmia. *AF* atrial fibrillation, *SVEB* supraventricular extrasystole, *SVRs* short supraventricular runs, *VEB* ventricular extrasystole.

All patients experienced arrhythmias after stroke. Among them, AF was detected in 5 (4%) patients, and it was asymptomatic in all cases. As regards as other rhythmic disturbances, bradycardia was observed in 7 (6%) individuals, supraventricular tachycardia in 25 (20%), SVEB in 116 (94%), SVRs in 67 (54%), and VEB in 108 (88%) (Fig. 1).

The patients with AF (AF group) underwent thrombolysis more frequently than patients without AF detection (no-AF group) (60 vs. 17%; p = 0.018). No correlation was found between NIHSS scores and AF (p = 0.26) and between AF and either SVRs (p = 0.27) or bradycardia (p = 0.52).

A sub-analysis compared all types of cardiac arrythmia detected at the first 24 h of 7-day Holter ECG to those reported during the whole 7-day ECG recording. 7-day Holter ECG monitoring proved to be superior as compared to 24-h recording for the detection of all arrhythmias (statistics is reported in Table 2).

**Table 2:** Comparison between frequency of detection of arrhythmic events by 7-day and 24-h continuous ECG monitoring.

	7-day Holter ECG	24-h monitoring	p-value
AF, n (%)	5 (4%)	0	0,0238
Bradycardia, n (%)	7 (6%)	1 (1%)	0,0310
Supraventricular tachycardia, n (%)	25 (20%)	5 (4%)	0,0001
SVEB, n (%)	116 (94%)	98 (80%)	0,0002
SVRs, n (%)	67 (54%)	21 (17%)	0,0001
VEB, n (%)	108 (88%)	83 (68%)	0,0001

*ECG* electrocardiogram, *n* number of patients, *AF* atrial fibrillation, *SVEB* supraventricular extrasystole, *SVRs* short supraventricular runs, *VEB* ventricular extrasystole.

None of the arrhythmic events, considered as dependent variable using repeated measures of ANOVA, showed any association with a prior history of hypertension, diabetes, coronary artery disease, heart failure, smoking, patent foramen ovale, or previous stroke.

**Figure 2:**
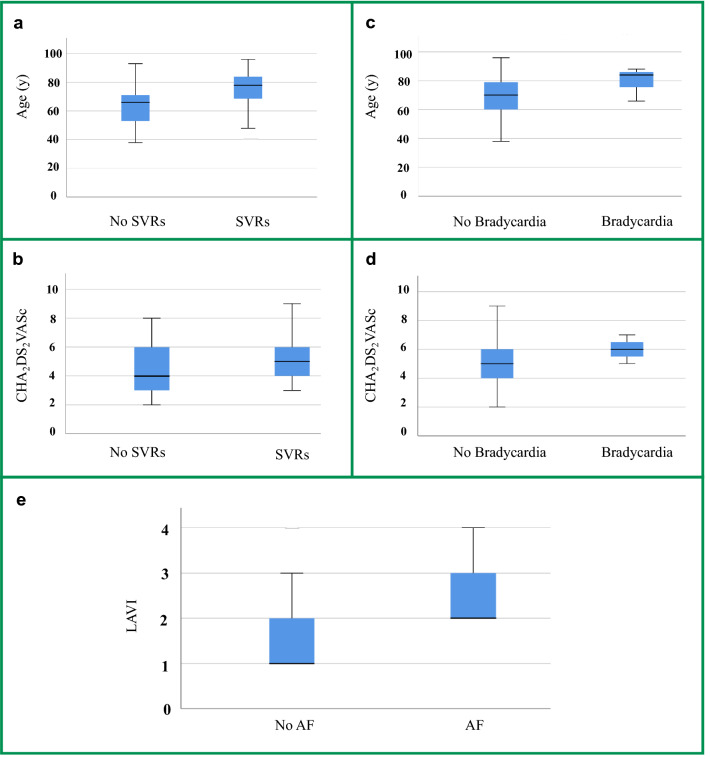
The Box Plots represent the comparisons among different cardiac arrythmias (SVRs, bradycardia, and AF), detected by 7-day Holter ECG, with age, CHA_2_DS_2_VASc score, and LAVI. Data are presented as mean ± standard deviation (SD). (**a**) Patients with SVRs were older (77.3 ± 13.3) compared to those without SVRs (66.5 ± 12.6), p = 0.0001; (**b**) patients with SVRs showed a higher CHA_2_DS_2_VASc score (5.3 ± 1.4) in comparison with the group without SVRs (4.0 ± 1.5), p = 0.004; (**c**) patients with bradycardia were older (83.1 ± 9.2) compared with those without bradycardia (69.8 ± 14.0), p = 0.035; (**d**) CHA_2_DS_2_VASc score was higher in patients with bradycardia (6.0 ± 0.8) in comparison with individuals without bradycardia (4.8 ± 1.5), p = 0.026; (**e**) A higher LAVI was detected in AF group compared to no-AF group (p = 0.002); 1 = normal LAVI (16–28 ml/m^2^), 2 = mildly abnormal (29–33 ml/m^2^), 3 = moderately abnormal (34–39 ml/m^2^), and 4 = severely abnormal (≥ 40 ml/m^2^). *SVRs* short supraventricular runs, *CHA*_*2*_*DS*_*2*_*VASc* score assessing risk of atrial fibrillation, *AF* atrial fibrillation, *LAVI* left atrial volume index.

In addition, patients with SVRs were older (77.3 ± 13.3 vs. 66.5 ± 12.6 years; p = 0.0001) and had higher CHA_2_DS_2_VASc scores (5.3 ± 1.4 vs. 4.0 ± 1.5; p = 0.004) compared to subjects without SVRs (Fig. 2a,b). CHA_2_DS_2_VASc score (6.0 ± 0.8 vs. 4.8 ± 1.5; p = 0.026) and age (83.1 ± 9.2 vs. 69.8 ± 14.0 years; p = 0.035) were also higher in patients with bradycardia (Fig. 2c,d). No significant results were found for the presence of SVEB, VEB, supraventricular tachycardia, and AF in relation to age and CHAD_2_VASC_2_ scores.

No significant difference was found in the detection rate of any type of arrhythmia, considering the time of 7-day Holter ECG placement (measured in days) after stroke.

### Transthoracic echocardiography

A higher LAVI was detected in AF group compared to no-AF group (p = 0.002) (Fig. 2e). No difference in ejection fraction was documented between the two groups.

### Site of ischemic lesion

The cerebral ischemic lesions were divided into the following categories: side (right or left), insular involvement (yes or not), size (lacunar or no-lacunar stroke), and site (brainstem, cerebellum, basal ganglia, insula, frontal cortex, parietal cortex, temporal cortex, occipital cortex, thalamus, corona radiata, and extensive lesion, when more than a region was involved).

Using repeated measures of ANOVA, each arrhythmic event was considered as a dependent variable compared to the site of ischemic lesion. A significant correlation was found among SVEB onset and parietal (25 vs. 2%; p = 0.013) and temporal (25 vs. 2%; p = 0.013) cortical lesions. Conversely, no associations were found between other arrhythmic events and the remaining sites of ischemic infarction. In addition, VEB occurrence correlated with insular involvement (64 vs. 33%; p = 0.002). No association was found for lacunar stroke and side of lesion compared to each cardiac arrhythmia.

## Discussion

The present study shows the rate of arrhythmias occurring after stroke, suggesting the importance of a strict monitoring after cerebrovascular events to prevent possible worse outcomes due to cardiological accidents. Our findings confirm also the higher sensitivity of 7-day Holter ECG, in the detection of all arrhythmias considered, compared to 24-h ECG recording^24^. As expected, the rate of arrhythmia detection increases with prolonged heart rhythm monitoring^24,25^ (see Table 2). Even if all our cases of AF were asymptomatic and characterized by a paroxysmal pattern, AF was identified in 4% of our patients using the 7-day Holter ECG. No AF episodes were found with 24-h ECG recording. Hence, our finding seems to be in accordance with previous literature, which showed that 7-day Holter ECG provides a higher AF detection rate, approximately up to 10–15% of cases^14,26^ as compared to nearly 2% in 24 h recording^24^. Therefore, the use of 7-day Holter ECG can represent a first-line approach in arrhythmia detection after stroke because of its low costs, effectiveness, and non-invasiveness^27^. A prolonged cardiac monitoring, such as 7-day Holter ECG, should be placed after stroke to detect rhythm disturbances for both therapeutical management and prognostic evaluation. For the well-known interaction between cerebrovascular events and cardiac disturbances^1,2^, this device should be considered in all individuals with diagnosis of acute stroke, and especially in those with a cryptogenic or embolic origin.

Newly diagnosed arrythmias are commonly observed after stroke, even if it is currently unclear whether they should be considered as cardiogenic or neurogenic events^28^.

As regards to the cardiogenic hypothesis, although the presence of AF is a well-established cause of stroke, other coexisting cardiological factors (e.g., endothelial dysfunction, fibrosis, and atrial abnormalities) seem to be associated with rhythm disturbances and, consequentially, thromboembolism^29^. Of interest in our study, LAVI was higher in AF group compared to no-AF group, confirming the previous assumption of the strict association between LAVI and AF^30^. As previously reported^20,31^, a higher LAVI is considered a well-known risk factor for AF and, therefore, it might be a worthwhile clinical predictor for identifying individuals with a major risk of AF and thromboembolic complications, optimizing both therapeutic management and outcomes^30,31^. Considering other cardiac disturbances, episodes of SVRs were found to correlate with age and CHA_2_DS_2_VASc score, in accordance with previous findings^32,33^. A similar association with age and CHA_2_DS_2_VASc score was also observed for bradycardia, which may be an expression of sinus node dysfunctions, such as in the Brady-Tachy Syndrome. These conditions are commonly associated with AF, suggesting the presence of a cardiac rhythm disturbance, which represents a risk factor for embolic ischemic strokes^34,35^. However, in our results, the lack of correlation between bradycardia and AF and between AF and either CHAD_2_VASC_2_ score or age might be explained by the small sample size of subject with AF (only 4% of the whole sample).

Hence, sinus and atrio-ventricular nodal diseases, including Brady-Tachy Syndrome, SVRs, and AF, might represent the result of either an underlying myocardial pathology (shown also by atrial abnormalities, such as a higher LAVI) or a central autonomic imbalance due to stroke^29^. Moreover, the copresence of cardiovascular risk factors, calculated for instance by CHA_2_DS_2_VASc score, may worsen the incidence of both cerebrovascular and cardiac accidents^2,32^.

As concerns the assumption of a CNS involvement in arrhythmogenesis, the association between cerebral infarction and cardiac events has been largely debated^12,36^. A pivotal role in autonomic regulation is represented by different cerebral structures, which may act after their damage in atrial remodelling and arrhythmia generation^28^. Because of its autonomic and limbic connections, converging evidences point toward the role of insula in VEB occurrence^13,37–39^, data confirmed in our results. As well as insula involvement, also temporal and parietal lobes have been related to the onset of severe cardiac arrhythmias^12,13^. Our findings showed a significant correlation among SVEB and temporal and parietal regions. A previous study showed as atrial premature beats were connected to parieto-temporal area in up to 20% of cases^40^. However, it remains unclear whether the effects of parietal infarction are directly due to a central autonomic dysfunction or it is necessary to consider parietal lobe only as a bystander region, which shares with the insula the same vascular supply^13^. Certainly, lesions of parietal cortex have been shown to be an independent predictor of both long-term cardiac morbidity and mortality^3,12,13^.

Limitations of our study are related to the sample size, which is not wide enough for generalisable considerations. Indeed, the low statistical power of our cohort did not allow to identify a strict association between all arrhythmias and cerebral infarctions and to support the hypothesis of a CNS role for arrhythmogenesis. Moreover, it might be useful to compare the rates of arrhythmias after stroke with those of a population of healthy controls to better evaluate the higher incidence of heart rhythm disturbances after stroke. Additional investigations are needed to confirm our findings.

## Conclusions:

Cardiac arrhythmias represent an increased risk factor for complications after stroke. Prolonged cardiac monitoring is a well-recognized strategy to identify individuals to submit to further cardiological examinations and therapies. For its technical characteristics (e.g., non-invasiveness and effectiveness) and its low costs, 7-day Holter ECG should be considered as a first-line approach. Therefore, 7-day Holter ECG should be placed in the first hours after stroke and in all subjects with cryptogenic stroke in order to put in place timely therapeutical strategies.

Further investigations are needed to deeply understand the correlation between cerebral ischemic lesions and the occurrence of arrhythmic events.

## Data Availability

The data are deposited in the SS Annunziata Hospital of Chieti repository.
